# Corrosion Behaviour of Injection- and Compression-Moulded Nd–Fe–B and Sm–Fe–N Magnets with Different Polymer Binders

**DOI:** 10.3390/polym18091123

**Published:** 2026-05-02

**Authors:** Nikolina Lešić, Nataša Kovačević, Ingrid Milošev

**Affiliations:** 1 Department of Physical and Organic Chemistry, Jožef Stefan Institute, Jamova cesta 39, 1000 Ljubljana, Slovenia; nikolinal93@gmail.com; 2 Jožef Stefan International Postgraduate School, Jamova cesta 39, 1000 Ljubljana, Slovenia; 3 Kolektor Mobility d.o.o., Vojkova ulica 10, 5280 Idrija, Slovenia; natasa.kovacevic@kolektor.com

**Keywords:** Nd–Fe–B, Sm–Fe–N, injection moulding, compression moulding, polymer-bonded magnets, corrosion resistance, magnetic properties, bulk corrosion test, thermal shock test

## Abstract

The corrosion behaviour and environmental durability of injection- and compression-moulded Nd–Fe–B and Sm–Fe–N magnets were investigated. For injection-moulded magnets, the effects of magnetic powder type (Nd–Fe–B and Sm–Fe–N), magnetic powder particle size (100 µm and 400 µm), and polymer binder (PPS and PA12) on corrosion resistance were studied. For compression-moulded magnets with an epoxy binder, the effects of powder type and size were examined. Corrosion resistance was investigated using potentiodynamic polarisation in electrolytes of varying pH (1.8–12.8). The Sm–Fe–N magnets exhibited slightly better corrosion resistance than the Nd–Fe–B magnets, irrespective of the polymer binder. The finer magnetic powders (100 µm) showed lower corrosion resistance due to their larger specific surface area, with a more pronounced effect in the compression-moulded magnets. The type of polymer binder had only a minor effect. The hygrothermal corrosion resistance and thermal stability were evaluated using bulk corrosion (BCT) and thermal shock tests, respectively. Surface corrosion was observed in all magnets after the BCT, with the compression-moulded magnets exhibiting a greater irreversible loss of magnetic properties. The thermal shock test caused a temporary reduction in magnetic properties, with recovery after remagnetisation, demonstrating the good thermal stability of both magnet types.

## 1. Introduction

Polymer-bonded magnets (PBMs), particularly rare-earth permanent magnets based on Nd–Fe–B and Sm–Fe–N, are widely used in engineering applications requiring strong magnetic performance combined with mechanical flexibility, complex geometries, and corrosion resistance, owing to their ease of processing and lower production costs compared to permanent metallic (sintered or hot pressed) magnets [1,2,3]. Therefore, these magnets find use in automotive applications, electronics, and medical devices [4,5]. In the bonded magnet process, hard magnetic particles are combined with a polymer binder at a defined mixing ratio (loading fraction) using a mixer or extruder. The obtained composite material is subsequently injection- or compression-moulded into bonded magnets [6,7]. Achieving optimal magnetic, mechanical, and corrosion properties in PBMs depends on the appropriate selection of magnetic material and polymer binder, as well as the dispersion of the magnetic particles within the polymer matrix [8,9,10].

Injection-moulded magnets are manufactured by injecting a mixture of magnetic powder and polymer, in the form of pellets produced by extrusion, into a mould [11,12]. Injection moulding offers great flexibility in shape design and high dimensional precision, and enables overmoulding and insert moulding with other components for easy and reliable assembly [9]. Thermoplastic polymers that are typically used in the injection moulding process include poly(*p*-phenylene sulphide) (PPS), polyamide 6 (PA6), polyamide 11 (PA11), and polyamide 12 (PA12) [13]. PPS polymer is a semi-crystalline high-performance polymer with excellent mechanical properties and high thermal stability, as well as low melt viscosity and moisture absorption [14,15,16]. Due to its highly crystalline regions, PPS shows excellent chemical resistance and remains insoluble in organic and inorganic solvents at temperatures below 200 °C [14,17]. PA12, also known as Nylon 12, is a semi-crystalline thermoplastic polymer characterised by lower moisture absorption than other polyamides while exhibiting comparable chemical resistance [18,19]. Owing to its mechanical flexibility, this polymer is commonly employed in the production of PBMs for applications requiring ductile behaviour, in contrast to PPS, which exhibits a more brittle character [12,20,21].

Compression-moulded magnets are manufactured by first mixing the magnetic powder with the polymer binder and then compressing the mixture in a press mould [11,22]. These magnets enable higher volume fractions of magnetic powder, resulting in superior magnetic performance compared to injection-moulded magnets [11,23]. Owing to their highly filled composite structure, compression-moulded magnets exhibit high stiffness and resistance to deformation [22]. Thermosetting polymers, such as epoxy, are usually employed in compression moulding [11]. Epoxy polymer exhibits low viscosity, high adhesive strength, favourable mechanical properties, and good chemical resistance [24,25]. It is commonly used in the industrial production of PBMs due to its ease of moulding and processing, in contrast to the more demanding processing techniques required for thermoplastic PBMs. However, inadequate corrosion resistance, insufficient high-temperature stability, and limited mechanical performance of epoxy-bonded magnets restrict their range of applications [26].

The polymer characteristics and its rheological behaviour, together with the magnetic powder properties (type, morphology, and particle size), determine the maximum achievable volume fraction of magnetic particles within the polymer matrix [12,27]. For PPS- and PA12-bonded magnets, these maximum volume fractions are approximately 65% and 70%, respectively [12,20,27]. The limiting operating temperatures of PPS and PA12 are approximately 220 °C and 150 °C, respectively [14,20,27]. In contrast, epoxy-bonded magnets can achieve substantially higher volume fractions than injection-moulded magnets, reaching approximately 85% [27,28], but their operating temperatures are generally limited to only around 125 °C [11,23].

Rare-earth PBMs are well known for their excellent magnetic properties [27,29], but their limited thermal stability and corrosion resistance, especially in humid or aggressive environments, remain critical limitations [10,30,31,32]. The reason for this is the material’s multiphase microstructure and a high concentration of rare-earth elements (a mass fraction of around 30% for Nd–Fe–B magnets), as well as insufficient wetting of the magnetic particles by polymer and the interaction between magnetic powder and polymer binder [5,33]. Although the multiphase microstructure provides favourable magnetic properties, it also contributes to the poor corrosion resistance of permanent magnets [34,35]. In PBMs, the presence of the polymer matrix improves corrosion resistance and mechanical properties, but magnetic performance is somewhat reduced compared to that of metallic permanent magnets due to the non-magnetic nature of the polymer binder [11,13,22].

Nd–Fe–B magnets represent the most significant class of rare-earth permanent magnets, owing to their superior energy density [13,36]. Sm–Fe–N magnets are known for their high Curie temperature, high saturation magnetisation, and large anisotropy field [37,38]. However, Sm–Fe–N magnets are less competitive compared to Nd_2_Fe_14_B magnets because they exhibit thermal instability at the temperatures required for the conventional high-temperature sintering and decompose into α-Fe and Sm–N phases above 600 °C [39].

The processability and performance of PBMs are governed by the magnetic powder type, shape, size, and concentration, as well as by the polymer properties and the adhesion quality between the magnetic powder and the polymer binder [9]. Spherical powder particles are generally preferred for injection-moulded magnets because their morphology improves flow behaviour, thereby reducing the viscosity of the powder-polymer melt [27,38].

Xiao and Otaigbe [10] investigated the roles of particle size and surface modification in the oxidation and corrosion behaviour of PPS-bonded Nd–Fe–B magnets. They found that applying a silane coupling agent to Nd–Fe–B powders improves resistance to oxidation and corrosion at elevated temperatures and 100% humidity, with the coating treatment having a more pronounced effect on smaller particles. In addition, silane-coated Nd–Fe–B powders were found to exhibit enhanced magnetic and mechanical properties owing to improved mixing and stronger interfacial adhesion between the polymer binder and the magnetic powder [8,9]. Consistent with these findings, similar improvements in mechanical and magnetic properties, together with oxidation and corrosion resistance, have also been reported for epoxy-bonded Nd–Fe–B magnets modified with silane-coated powders [40,41].

In another study, Otaigbe et al. [42] investigated the influence of coupling agent and magnetic powder size on the melt rheological behaviour of PPS-bonded Nd–Fe–B magnets. They found that minimum viscosity of PBMs around 290 °C was achieved using silane-coated Nd–Fe–B powders with particle sizes in the 106–150 μm range.

Handstein et al. [43] studied the impact of particle size on the magnetic behaviour of Nd–Fe–B polymer-bonded magnets. They reported a reduction in the intrinsic coercivity of PBMs with decreasing particle size, attributed to oxide phases that facilitate nucleation of internal demagnetising processes. A simultaneous decrease in remanence was also observed as the particle size decreased.

The effect of PPS and PA polymers on the mechanical properties of Nd–Fe–B polymer-bonded magnets has also been investigated. Garrell et al. [12,44] reported that PPS-bonded magnets exhibit higher mechanical strength than PA-bonded magnets, especially at elevated temperatures. Failure at higher temperatures was primarily attributed to interfacial debonding between the Nd–Fe–B particles and the PPS matrix. Superior mechanical performance of PPS-bonded magnets compared to PA-bonded magnets has also been demonstrated for additively manufactured (3D-printed) Nd–Fe–B polymer-bonded magnets [20,45].

Devices incorporating PBMs operate under a wide range of service conditions, including exposure to different media with varying pH values, as well as broad and often rapid changes in temperature and humidity [34]. Understanding the behaviour of magnets under different conditions is essential for selecting and designing PBMs to achieve optimal properties for a specific application. In our previous study [46], the corrosion behaviour of the NdFeB-400-PPS and SmFeN-400-PPS magnets was discussed in detail. However, a systematic understanding of the combined influence of magnetic powder type, particle size, and polymer binder on the corrosion behaviour of PBMs under different environmental conditions remains lacking. In this study, the corrosion behaviour of Nd–Fe–B and Sm–Fe–N polymer-bonded magnets produced by injection and compression moulding was investigated. The focus was placed on the effect of magnetic powder type (Nd–Fe–B and Sm–Fe–N), magnetic powder particle size (100 µm and 400 µm), and polymer binder of the injection-moulded magnets (PPS and PA12) on the corrosion resistance of the magnets, providing a systematic evaluation of these parameters. This study combines electrochemical measurements with environmental testing to evaluate the combined effects of these parameters on corrosion behaviour and their impact on the practical performance of the magnets under service-relevant and accelerated environmental conditions.

## 2. Materials and Methods

### 2.1. Materials, Sample Preparation, and Chemicals

The polymer-bonded Nd–Fe–B and Sm–Fe–N magnets in disc and block forms were used as substrates. The magnets were manufactured by Kolektor Mobility d.o.o., Idrija, Slovenia, using injection-moulding and compression-moulding processes. The processing parameters were selected in accordance with standard industrial practice to ensure adequate mechanical and magnetic properties of the PBMs. Specific production parameters are not reported, as they are proprietary to the manufacturer. However, all relevant experimental details are provided. The magnets produced by injection moulding contained the polymer binder PPS or PA12 at a volume fraction of 40%, whereas those produced by compression moulding contained epoxy as the polymer binder at a volume fraction of 20%. Two isotropic MQP-B+ magnetic powders (Magnequench (Tianjin) Co., Ltd., Tianjin, China) were used for the production of Nd–Fe–B magnets. The first powder was classified as 40-mesh, indicating that all particles were sieved through a mesh with openings of approximately 400 μm, although the actual median particle size (D50) was significantly smaller, around 160 μm, as reported in the manufacturer’s datasheet. The second powder was a finer 150-mesh grade, corresponding to particles smaller than ≈100 μm, with a reported D50 of approximately 65 μm. An isotropic SFN3H magnetic powder (Daido Electronics Co., Ltd., Gifu, Japan) was used for the production of Sm–Fe–N magnets. This powder was also designated as 40-mesh, with particle sizes smaller than ≈400 μm, but had a D50 of approximately 95 μm, indicating a significantly finer particle size than implied by the mesh classification alone. It should be noted that the mesh size refers to the sieve aperture through which the powder has passed, i.e., the maximum particle size that can pass through the sieve, not to the actual particle size, which depends on the material and processing method and is typically much smaller. The polymer-bonded magnets used in this study, together with their production process, magnetic powder type and particle size, polymer binder, and abbreviations used throughout the text, are listed in Table 1. A total of nine magnets of different combinations were investigated.

For electrochemical measurements, the samples were prepared through water-based grinding with P320–P4000 grit SiC papers (Struers, Ballerup, Denmark). Following grinding, the samples were rinsed with Milli-Q water, ultrasonically cleaned in absolute ethanol (Carlo Erba Reagents, Cornaredo, Milan, Italy; purity ≥ 99.9%) for 2 min, rinsed once more with Milli-Q water, and then dried using a nitrogen stream. For environmental testing, the samples were prepared using the same procedure, except that water-based grinding was performed only with P320 grit SiC paper.

Six electrolytes differing in pH were employed: 0.01 M H_2_SO_4_ (pH 1.8), 0.1 M NaCl (pH 5.6), 0.001 M Na_2_SO_4_ (pH 6.6), borate buffer (pH 9.3), 0.0001 M NaOH (pH 9.9), and 0.1 M NaOH (pH 12.8). These electrolytes were selected to span a broad pH range, reflecting the wide range of applications of PBMs and their potential exposure to different ionic species (sulphate, chloride, hydroxyl) during transport, storage, or service. All electrolytes were prepared using analytical-grade reagents: H_2_SO_4_ (Carlo Erba Reagents, Cornaredo, Milan, Italy; purity 96%), NaCl (Fisher Chemical, Pittsburgh, PA, USA; purity ≥ 99.5%), Na_2_SO_4_ (Acros Organics, Geel, Belgium; purity ≥ 99.0%), and NaOH (Labochem International, Athens, Greece; p.a.). The borate buffer solution was prepared from NaOH and Na_2_B_4_O_7_·10H_2_O (Honeywell Fluka, Charlotte, NC, USA; purity ≥ 99.0%). The pH values of the prepared electrolytes were determined using an 827 pH Lab pH meter (Metrohm AG, Herisau, Switzerland) equipped with a Metrohm Unitrode^®^ electrode (Pt1000, *c*(KCl) = 3 M).

The water used for sample rinsing and electrolyte preparation was obtained from a Milli-Q^®^ Direct Water Purification System (Merck Millipore, Billerica, MA, USA), providing ultrapure water with a resistivity ≥ 18.2 MΩ cm at 25 °C.

### 2.2. Microstructural and Chemical Characterisation

SEM/EDS analysis was employed for the surface morphology and elemental characterisation of three different magnetic powders used in polymer-bonded magnet production in our research, i.e., Nd–Fe–B MQP-B+ (≈400 μm), Nd–Fe–B MQP-B+ (≈100 μm), and Sm–Fe–N SFN3H (≈400 μm). A JSM-IT300 scanning electron microscope (JEOL Ltd., Tokyo, Japan), equipped with an Oxford X-Max^N^ (SDD 80 mm^2^) EDS detector (Oxford Instruments plc, Abingdon, UK), was used. SEM micrographs were acquired in low-vacuum mode employing a backscattered electron detector (BED-S) at an accelerating voltage of 15 kV, with the working distance set between 10.8 mm and 13.0 mm to maximise image resolution and compositional contrast. EDS area analysis was performed at multiple locations on the powders, as indicated by the rectangles, and the elemental compositions are reported in atomic per cent (at. %).

### 2.3. Electrochemical Measurements

To investigate the corrosion properties of the polymer-bonded magnets, potentiodynamic polarisation (PDP) curves were recorded using a Multi Autolab/M204 potentiostat/galvanostat (Metrohm Autolab, Utrecht, The Netherlands), which was operated with Nova 2.1.4 software. All measurements were conducted at room temperature in a 250 mL custom-built three-electrode cell. The samples were positioned laterally within the electrochemical cell and held in position with a rubber band, with electrical contact established on the opposite side using copper tape. A graphite rod and an Ag/AgCl (sat. KCl) electrode (*E*_Ag/AgCl_ = 0.197 V vs. SHE) served as the counter and reference electrodes, respectively. All potentials reported in this work are referenced to the Ag/AgCl electrode. To ensure reproducibility, each measurement was performed at least three times, and representative PDP curves are shown.

The PDP measurements were performed in six electrolytes, as described in Section 2.1. Prior to each measurement, the open-circuit potential (OCP) was monitored for 1 h. The polarisation scans were recorded from −0.25 V below the OCP to various anodic potentials, depending on the magnet under investigation (ranging from 0.8 V to 2 V), at a scan rate of 1 mV s^−1^. Electrochemical parameters, including the corrosion current density (*j*_corr_) and the corrosion potential (*E*_corr_), were obtained using the Tafel extrapolation method. The assessment of electrochemical parameters was carried out according to the rule that the linear portion of the Tafel plot should cover one order of magnitude in current density, if possible two, to ensure reliable extrapolation [47]. In cases where this requirement was not satisfied, the *j*_corr_ was evaluated from the intersection of the fitted linear portion of the cathodic branch and the line corresponding to the *E*_corr_. The *j*_corr_ and *E*_corr_ values are reported as the mean values of several repeated measurements along with their associated standard deviations. For passivated samples, the primary passivation potential (*E*_pp_) defines the onset of the passive region. In contrast, the breakdown potential (*E*_br_) corresponds to the potential at which a rapid increase in current density occurs, indicating the termination of passivity. The passive region (Δ*E*_pass_) is defined as the potential range between the *E*_pp_ and the *E*_br_. For the electrochemical measurements, the magnetic materials were not magnetised to avoid magnetic field effects.

### 2.4. Environmental Testing

To assess the environmental durability of the Nd–Fe–B and Sm–Fe–N polymer-bonded magnets, the bulk corrosion test (BCT) and the thermal shock test were performed as part of environmental testing to simulate harsh ambient conditions. A total of nine magnets (Table 1) were tested using each method, with two samples of each magnet type tested simultaneously to achieve reproducibility.

The bulk corrosion test was conducted under controlled atmospheric conditions in accordance with ASTM A1071/A1071M-11 standard [48]. The aim was to determine the corrosion resistance of the magnets under high-temperature and high-pressure water vapour conditions. During the test, the samples were exposed to a saturated steam environment (100% RH) at 120 ± 2 °C for 96 h. At this temperature, the nominal steam pressure was approximately 2 bar.

The thermal shock test was conducted in air in accordance with DIN EN 60068-2-14 standard [49]. The objective was to evaluate the ability of the polymer-bonded magnets to endure rapid variations in ambient temperature. During the test, the samples were cyclically transferred between cold and hot air chambers (−40 °C and +140 °C), with a 30 min dwell time in each chamber, for a total of 600 cycles. The transition duration time between the chambers was less than 30 s.

After both tests, microstructural observations of the samples were performed using an Olympus BX51 optical microscope (Olympus Corporation, Tokyo, Japan) in the bright-field mode. The total magnification was 100×, obtained using a 10× objective lens (Olympus UMPlanFI 10×/0.30 BD) and a 10× eyepiece (WHN 10×/22).

High-resolution X-ray computed tomography (HRXCT) scanning was employed to investigate the internal structural changes in the samples following the bulk corrosion test and the thermal shock test. Scanning was performed with a GE Phoenix v|tome|x s240 system (General Electric, Cincinnati, OH, USA). The system was operated at 180 kV and 250 µA, with an exposure time of 200 ms per projection. This non-destructive technique enabled the visualisation and analysis of internal features such as pores, cracks, and material degradation in three dimensions.

To investigate the effects of environmental tests on the magnetic properties of the Nd–Fe–B and Sm–Fe–N polymer-bonded magnets, the magnetic dipole moment (*m*) was measured before and after both the bulk corrosion test and the thermal shock test. For the thermal shock test, the magnetic dipole moment was additionally measured after 300 cycles, after which the samples were returned to the chamber, and the test was continued to a total of 600 cycles. Since the samples were magnetised to saturation prior to testing, changes in magnetic dipole moment could be directly monitored. Additionally, after both tests, the samples were remagnetised to saturation, and the magnetic dipole moment was measured again to determine whether the losses after the tests were due to reversible or irreversible changes in the material. Magnetisation of the samples was performed using an IMD-2500-3300-60KA-e impulse magnetiser (Stute Magnet Technik, Schwerte, Germany) at an applied voltage of 2000 V, ensuring magnetic saturation. Magnetic dipole moment measurements were carried out using a fluxmeter F10 in conjunction with a Helmholtz coil HC 150 (both from Brockhaus Messtechnik GmbH & Co. KG, Lüdenscheid, Germany). The Helmholtz coil consisted of two identical circular coils positioned parallel to each other at a distance equal to their radius (75 mm), forming a Helmholtz configuration that generates a relatively uniform magnetic field in the central region. By placing a magnet at the centre of the coil system and measuring the change in magnetic flux as the magnet is removed from the coil, a fluxmeter can determine the magnetic dipole moment of the magnet. All measurements were conducted at room temperature and repeated several times for each sample to ensure reproducibility. The results are presented in graphs, where each data point represents the mean value of two samples tested for each material in both the bulk corrosion and thermal shock tests, along with the corresponding standard deviations.

## 3. Results and Discussion

### 3.1. Microstructural and Chemical Characterisation

**Figure 1:**
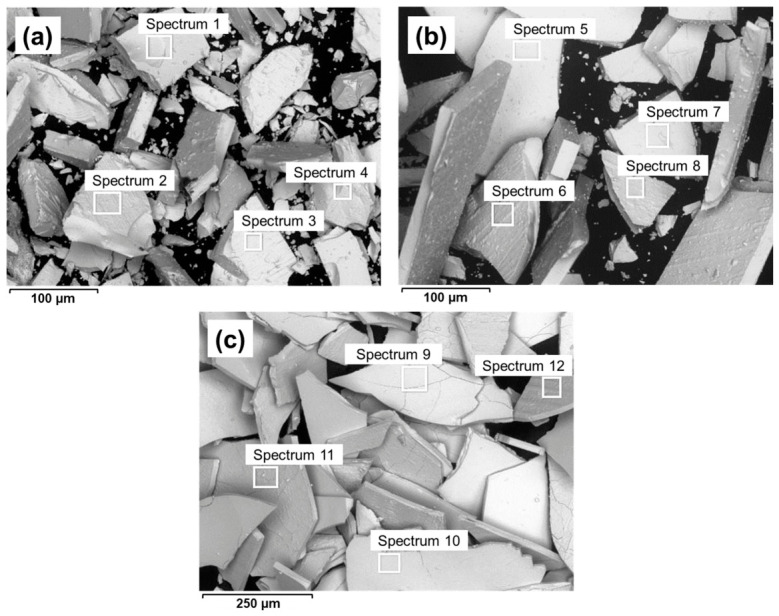
SEM images of (**a**) Nd–Fe–B MQP-B+ (100 μm), (**b**) Nd–Fe–B MQP-B+ (400 μm), and (**c**) Sm–Fe–N SFN3H (400 μm) magnetic powders. The corresponding EDS analysis for the numbered sites is presented in Table 2.

To investigate the effects of both magnetic powder type and particle size on the corrosion behaviour of the Nd–Fe–B and Sm–Fe–N polymer-bonded magnets, three different powders were used: Nd–Fe–B MQP-B+ with a particle size of 100 μm, Nd–Fe–B MQP-B+ with a particle size of 400 μm, and Sm–Fe–N SFN3H with a particle size of 400 μm. The SEM images of these powders are shown in Figure 1. The EDS results, presented as mean compositions (at. %), are summarised in Table 2, whereas detailed EDS results are provided in Table S1.

All three magnetic powders have plate-like-shaped particles. The SEM analysis reveals that the MQP-B+ magnetic powder particles are significantly thicker than those of the SFN3H powder. In addition, the SFN3H powder appears to be much more brittle, which may be attributed either to the powder production process or to the intrinsic properties of the material (the material being nitride-based rather than boride-based). According to the manufacturer’s data, the Sm–Fe–N powder exhibits approximately twice the hardness of the Nd–Fe–B powder, as measured by micro-Vickers testing.

The EDS analysis revealed that the MQP-B+ magnetic powders consist predominantly of Nd, Fe, and B, with minor amounts of Co and trace amounts of Zn and Si, while additional content of Al was detected in the MQP-B+ (100 μm) powder. The composition of the Nd–Fe–B magnetic powder does not differ significantly depending on the size of the magnetic powder particles (100 and 400 µm) because it is the same type of magnetic powder, i.e., MQP-B+ powder, which is based on a Nd–Fe–Co–B composition. The SFN3H magnetic powder contains Sm, Fe, and N as the main elements, along with smaller amounts of Co, Al, Zr, and Ga. Both powders also exhibit elevated amounts of C and O, which may be attributed to oxidation, a process difficult to avoid during powder production, as well as possible contamination.

### 3.2. Electrochemical Measurements

**Figure 2:**
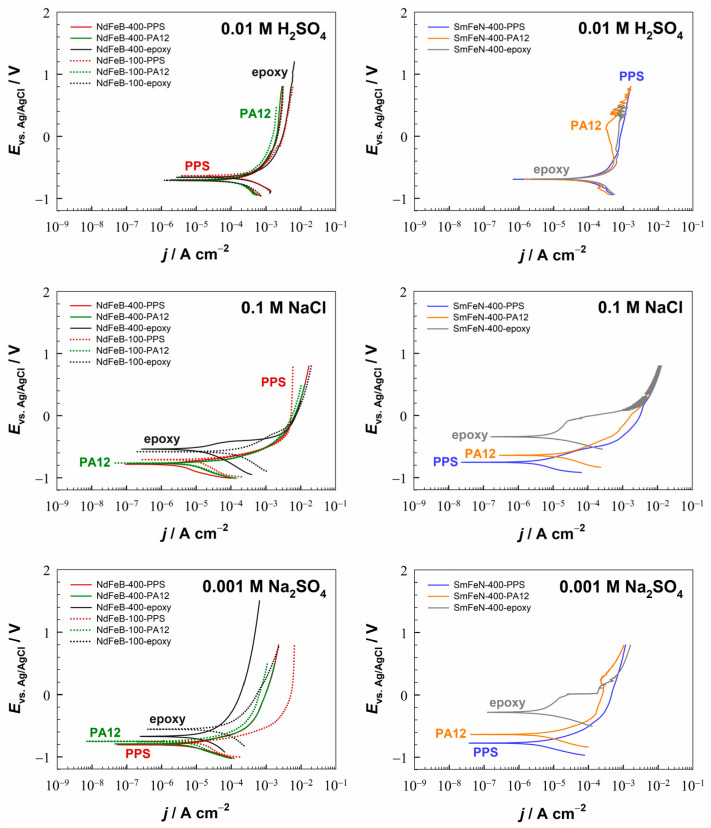
Potentiodynamic polarisation curves of Nd–Fe–B and Sm–Fe–N polymer-bonded magnets in 0.01 M H_2_SO_4_ (pH 1.8), 0.1 M NaCl (pH 5.6), and 0.001 M Na_2_SO_4_ (pH 6.6) electrolytes. Representative PDP curves obtained from measurements repeated under identical conditions are displayed. Before polarisation, the samples were allowed to stabilise at OCP for 1 h. Scan rate: 1 mV s^−1^; room temperature.

**Figure 3:**
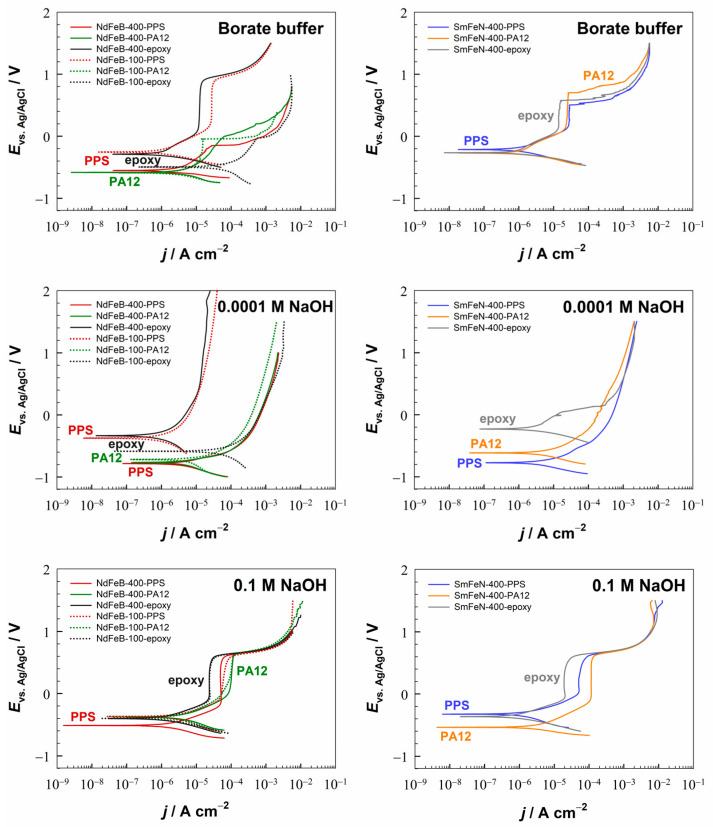
Potentiodynamic polarisation curves of Nd–Fe–B and Sm–Fe–N polymer-bonded magnets in borate buffer (pH 9.3), 0.0001 M NaOH (pH 9.9), and 0.1 M NaOH (pH 12.8) electrolytes. Representative PDP curves obtained from measurements repeated under identical conditions are displayed. Before polarisation, the samples were allowed to stabilise at OCP for 1 h. Scan rate: 1 mV s^−1^; room temperature.

**Figure 4:**
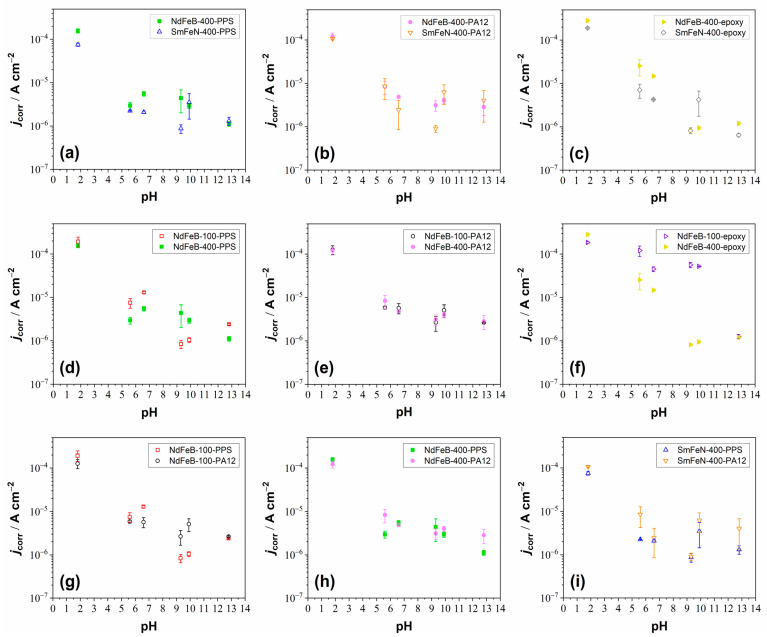
Graphs illustrating the influence of (**a**–**c**) magnetic powder type, (**d**–**f**) magnetic powder size, and (**g**–**i**) polymer binder on the corrosion behaviour of Nd–Fe–B and Sm–Fe–N polymer-bonded magnets, showing the dependence of *j*_corr_ on pH. The data were obtained from the PDP measurements and correspond to the mean values of several repeated measurements, with error bars representing the standard deviations.

Potentiodynamic polarisation measurements were conducted to examine the electrochemical response of the Nd–Fe–B and Sm–Fe–N polymer-bonded magnets in electrolytes with pH values between 1.8 and 12.8 at room temperature (Figure 2 and Figure 3). For easier comparison of materials, electrochemical parameters derived from the PDP curves, i.e., corrosion current densities (*j*_corr_) and corrosion potentials (*E*_corr_), are plotted as a function of pH (Figure 4 and Figure S1). The OCP curves of the NdFeB-400-PPS and SmFeN-400-PPS magnets, recorded over 1 h in all six investigated electrolytes, are presented in Figure S2.

In our previous study [46], the corrosion behaviour of the NdFeB-400-PPS and SmFeN-400-PPS magnets was discussed, together with the SEM/EDS and XPS analyses. It was suggested that in the 0.01 M H_2_SO_4_ electrolyte, corrosion products such as iron, neodymium, and/or samarium oxides/hydroxides/sulphates are formed, while in the 0.1 M NaCl electrolyte, corrosion products can include iron, neodymium, and/or samarium oxides/hydroxides/chlorides. In the 0.1 M NaOH electrolyte, passive films consisting of iron, neodymium, and/or samarium oxides/hydroxides are formed.

This study investigates the influence of various factors on the corrosion behaviour of Nd–Fe–B and Sm–Fe–N polymer-bonded magnets. Only meaningful comparisons were made; specifically, injection-moulded magnets were not compared with compression-moulded magnets, as these involve different processing methods that introduce additional variables affecting the materials. Moreover, the ratio of magnetic powder to polymer differs between injection-moulded and compression-moulded magnets, further justifying separate comparisons. Therefore, comparisons were limited to injection-moulded magnets with other injection-moulded magnets and compression-moulded magnets with other compression-moulded magnets.

The OCP monitoring of the NdFeB-400-PPS magnet indicates the presence of three distinct electrochemical regimes (Figure S2). An active dissolution regime is observed in acidic (H_2_SO_4_), near-neutral (Na_2_SO_4_ and NaCl), and weakly alkaline (0.0001 M NaOH) electrolytes, where OCP converges towards values below −0.7 V, indicating the dissolution of the native oxide layer and resulting in sustained surface activation. The continuous decline of *E*_OCP_ in the 0.0001 M NaOH suggests that the low concentration of OH^−^ ions is insufficient to maintain or repair the native oxide layer or to promote the formation of a stable passive film over time, eventually leading to a surface state similar to that in non-passivating acidic and near-neutral electrolytes. A metastable passive state is identified in the borate buffer, where the initially high potential is interrupted by sudden drops, likely associated with local breakdown followed by temporary reformation of the passive film. The passive regime is most pronounced in the 0.1 M NaOH, characterised by a continuous noble shift in OCP, indicating the formation and growth of a stable, protective passive film.

In contrast to the NdFeB-400-PPS magnet, the SmFeN-400-PPS magnet exhibits improved electrochemical stability, particularly in buffered and strongly alkaline electrolytes. In acidic (H_2_SO_4_) and near-neutral (Na_2_SO_4_ and NaCl) electrolytes, OCP shifts to active values below −0.65 V, indicating sustained dissolution of the native oxide layer. A pronounced downward drift is observed in the 0.0001 M NaOH, again suggesting progressive surface activation due to insufficient OH^−^ concentration to stabilise the surface oxide. In the borate buffer, the SmFeN-400-PPS reaches the most noble and stable potential, approximately −0.2 V, showing no evidence of the metastable breakdown observed in the NdFeB-400-PPS magnet. In the 0.1 M NaOH, a clear passivation process is observed, characterised by an initial positive shift in OCP followed by stabilisation at more noble potentials, indicating the formation of a stable protective passive film.

The PDP measurements showed that all magnets, regardless of polymer binder, magnetic powder particle size, or magnetic material, corroded in acidic (H_2_SO_4_) and near-neutral (Na_2_SO_4_ and NaCl) electrolytes, whereas they passivated in alkaline electrolytes (borate buffer and NaOH). With increasing pH, the *j*_corr_ values decreased from the order of 10^−4^ A cm^−2^ in acidic to 10^−7^ A cm^−2^ in alkaline media (Figure 4). The *E*_corr_ values increased slightly with increasing pH, ranging between −0.8 V and −0.2 V (Figure S1).

In the 0.01 M H_2_SO_4_ electrolyte (Figure 2), the differences between the magnets were minor. However, the SmFeN-400-PA12 magnet showed a slight decrease in the anodic current between −0.4 V and 0.2 V. Although such behaviour has not been observed for the Nd–Fe–B and Sm–Fe–N magnets with PPS, it has been reported [46] for the Nd and Sm metals and attributed to the possible formation of Nd/Sm sulphates that temporarily protect the metal surface. A similar mechanism may also be occurring in this case. In the 0.1 M NaCl and 0.001 M Na_2_SO_4_ electrolytes (Figure 2), the corrosion current densities were more than one order of magnitude lower than those measured in sulphuric acid, except for the NdFeB-100-epoxy magnet, which exhibited considerably higher *j*_corr_ values than the other magnets.

In alkaline media (borate buffer and NaOH), all magnets exhibited passivation, with the *j*_corr_ values of the order of 10^−6^ A cm^−2^ or 10^−7^ A cm^−2^. The only exception was the NdFeB-100-epoxy magnet, which showed relatively high *j*_corr_ values in mildly alkaline conditions and exhibited passivation only in the 0.1 M NaOH electrolyte. In the borate buffer (Figure 3), the NdFeB-400-epoxy magnet exhibited the widest passive region, approximately 800 mV, with the *E*_br_ of 900 mV. The NdFeB-100-PPS magnet also had *E*_br_ = 900 mV, and the width of the passive region was 700 mV. The NdFeB-100-PA12 magnet displayed a passive region of 300 mV, with the *E*_br_ of −100 mV. The SmFeN-400-PPS, SmFeN-400-PA12, and SmFeN-400-epoxy magnets all had *E*_pp_ = 100 mV, and the corresponding breakdown potentials were 500 mV, 700 mV, and 600 mV, respectively. Although the NdFeB-400-PPS and NdFeB-400-PA12 magnets did not display a clear active–passive transition, as observed for the other magnets, they showed a steeper slope of the anodic branch of the PDP curves, while the *j*_corr_ values were of the order of 10^−6^ A cm^−2^, indicating passivation. In the 0.0001 M NaOH electrolyte (Figure 3), only the NdFeB-400-epoxy magnet showed a broad passive region of approximately 1.5 V and the *E*_br_ of 1.8 V. Although the anodic branches of the PDP curves for the NdFeB-100-PPS, SmFeN-400-PPS, and SmFeN-400-epoxy magnets displayed steeper slopes, a distinct active–passive transition was absent. This absence of a well-defined passive region is consistent with the surface instability observed during the OCP monitoring (Figure S2), where the continuous downward drift toward more negative potentials indicates a progressive surface activation rather than stable passivation. Nevertheless, all magnets in the 0.0001 M NaOH electrolyte showed relatively low *j*_corr_ values, and their *E*_corr_ values were higher than those in acidic and near-neutral electrolytes. This suggests that, although this alkaline environment provides a baseline level of protection and results in partial passivation compared to more aggressive media, the passive state remains metastable and does not develop into a stable long-term protective film under anodic polarisation. In contrast, in the 0.1 M NaOH electrolyte (Figure 3), all magnets were passivated, showing a clearly defined passive region and a breakdown potential of approximately 600 mV, while the widths of the passive regions differed slightly. The NdFeB-400-PPS and SmFeN-400-PA12 magnets displayed Δ*E*_pass_ = 800 mV; the NdFeB-400-PA12 and SmFeN-400-PPS magnets exhibited Δ*E*_pass_ = 600 mV, whereas all the other magnets had Δ*E*_pass_ = 700 mV.

#### 3.2.1. The Influence of the Magnetic Powder Type

The influence of the type of magnetic powder, i.e., Nd–Fe–B and Sm–Fe–N, with 400 µm magnetic powder size, on the corrosion behaviour of the magnets is shown in Figure 2, Figure 3 and Figure 4a–c. Three comparisons are presented in terms of *j*_corr_ values. The first two comparisons are between injection-moulded magnets NdFeB-400-PPS and SmFeN-400-PPS (Figure 4a) as well as NdFeB-400-PA12 and SmFeN-400-PA12 (Figure 4b). The third comparison is between compression-moulded Nd–Fe–B and Sm–Fe–N magnets with epoxy as the polymer binder, i.e., NdFeB-400-epoxy and SmFeN-400-epoxy (Figure 4c). The presentation of *E*_corr_ values is given in the Supplement, Figure S1a–c. In general, the *j*_corr_ values (Figure 4a–c) decrease with increasing electrolyte pH, and the *E*_corr_ values (Figure S1a–c) shift to more positive values.

Injection-moulded magnets behave similarly across different electrolytes, with a minor difference: the Sm–Fe–N magnet showed slightly lower *j*_corr_ values than the Nd–Fe–B magnet in near-neutral and slightly alkaline electrolytes (Figure 4a,b).

Compression-moulded magnets exhibit a similar trend, with *j*_corr_ values decreasing across all electrolytes (Figure 4c). In all cases, except at pH 10, the values were slightly lower for the SmFeN-400-epoxy magnet than for the NdFeB-400-epoxy magnet.

The *E*_corr_ values of the Sm–Fe–N magnets were either similar or shifted toward more noble potentials compared with those of the Nd–Fe–B magnets for both injection- and compression-moulded magnets (Figure S1a–c). Overall, the results indicate that the Sm–Fe–N magnets exhibit slightly better corrosion resistance than the Nd–Fe–B magnets, regardless of which polymer binder was used.

#### 3.2.2. The Influence of the Magnetic Powder Size

The influence of the particle size of magnetic powder, i.e., 100 µm and 400 µm, on the corrosion behaviour of the Nd–Fe–B magnet is shown in Figure 2, Figure 3, Figure 4d–f and Figure S1d–f. The smallest differences in *j*_corr_ and *E*_corr_ values were detected for the magnets containing the PA12 polymer (Figure 4e and Figure S1e).

Slightly larger differences were observed for the magnets containing the PPS polymer (Figure 4d). Although the differences are minor, the magnet with a particle size of 100 µm exhibited higher *j*_corr_ values in acidic, near-neutral, and highly alkaline electrolytes, indicating greater corrosion susceptibility. In contrast, at pH 9–10, the same powder size showed lower *j*_corr_ values than the 400 µm magnetic powder. The *E*_corr_ values of the magnet with the 100 µm magnetic powder size were consistently shifted toward more positive potentials across all electrolytes (Figure S1d).

The largest differences were observed for the compression-moulded magnets containing the epoxy polymer (Figure 4f). This can be ascribed to the higher magnetic powder content in the compression-moulded magnets (volume fraction of 80%) compared to the injection-moulded magnets with PPS and PA12 polymers, thereby amplifying the observed effect. In this comparison, the magnet with the 100 µm powder exhibited higher *j*_corr_ values in all electrolytes, excluding strongly acidic and strongly alkaline media, indicating reduced corrosion resistance. The *E*_corr_ values were mostly shifted toward more negative potentials than those observed with the 400 µm magnetic powder, further supporting the lower corrosion resistance of the magnet with the 100 µm powder size (Figure S1f).

In general, considering the effect of particle size in the Nd–Fe–B magnets, somewhat lower corrosion resistance was observed for the 100 µm magnetic powder, with the extent of the difference varying slightly depending on the polymer binder. Lower corrosion resistance is likely associated with the larger specific surface area (defined as the total surface area of a material per unit of mass) of finer powders, which increases their susceptibility to corrosion [10]. Smaller particles have a higher surface-to-volume ratio, resulting in a higher specific surface area. Moreover, smaller differences between the magnets with two particle sizes were observed for the injection-moulded magnets, which may result from better interaction between the polymer and the magnetic powder, likely influenced by both the polymer type and the manufacturing method. Enhanced mixing during injection moulding improves encapsulation of the powder particles, providing greater protection and reducing the effect of particle size on the corrosion behaviour of magnets. In contrast, the compression-moulded magnets may exhibit insufficient mixing between the polymer and the magnetic powder, resulting in less effective encapsulation of the magnetic particles. As a consequence, the influence of particle size on the corrosion behaviour was more pronounced. Therefore, the corrosion resistance is influenced by both the manufacturing method and the polymer fraction.

#### 3.2.3. The Influence of the Polymer Binder

The influence of the polymer binder, i.e., PPS and PA12, on the corrosion behaviour of the Nd–Fe–B and Sm–Fe–N magnets is shown in Figure 2, Figure 3, Figure 4g–i and Figure S1g–i. The comparison was limited to the injection-moulded magnets, as they were produced using the same manufacturing method. Therefore, the comparisons were made between NdFeB-100-PPS and NdFeB-100-PA12, NdFeB-400-PPS and NdFeB-400-PA12, as well as SmFeN-400-PPS and SmFeN-400-PA12 magnets.

In all three cases, the *j*_corr_ and *E*_corr_ values of the magnets with different polymer binders were very similar, with the smallest differences observed between the NdFeB-400-PPS and NdFeB-400-PA12 magnets (Figure 4h and Figure S1h). The comparison between the NdFeB-100-PPS and NdFeB-100-PA12 magnets (Figure 4g) revealed slightly higher *j*_corr_ values for the PPS-based magnet in acidic and near-neutral media, whereas in alkaline media, the values were lower than those for the PA12-based magnet. For the SmFeN-400-PPS and SmFeN-400-PA12 magnets (Figure 4i), the latter showed slightly higher *j*_corr_ values across all electrolytes.

Nevertheless, the electrochemical measurements indicated no significant differences in the corrosion behaviour of the investigated materials, suggesting that, at least for PPS and PA12, the polymer binder has only a limited influence on the corrosion resistance of the magnets under the tested conditions. This behaviour may be attributed to the use of an identical manufacturing method, namely injection moulding, for their production and to the fact that they contain the same volume fraction of magnetic powder, which is the corrosion-prone component of the composite. In contrast, the performance of these magnets in high-temperature applications depends on the polymer binder. Specifically, the PA12 polymer has a lower heat deflection temperature than the PPS polymer and softens at elevated temperatures, thereby weakening the interfacial interaction between the polymer binder and the magnetic powder particles [14,18]. Therefore, PPS-based magnets are more suitable for high-temperature applications.

### 3.3. Environmental Testing

#### 3.3.1. Bulk Corrosion Test (BCT)

**Figure 5:**
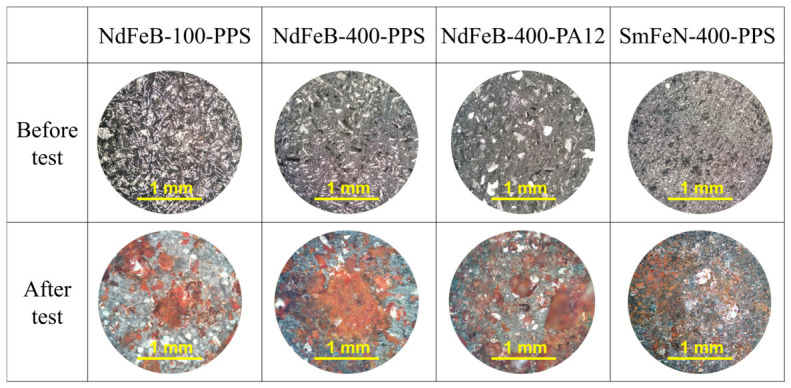
Optical microscopy images of Nd–Fe–B and Sm–Fe–N polymer-bonded magnets before and after the bulk corrosion test, taken in the bright-field mode at a total magnification of 100×. The BCT test conditions: 100% RH, 120 ± 2 °C, 2 bar, 96 h.

The bulk corrosion test was conducted to determine the resistance of the Nd–Fe–B and Sm–Fe–N polymer-bonded magnets to degradation caused by simultaneous exposure to high temperature and pressurised water vapour. The optical microscopy images of the NdFeB-100-PPS, NdFeB-400-PPS, NdFeB-400-PA12, and SmFeN-400-PPS magnets before and after the test are shown in Figure 5. Corrosion was observed in all magnets, with slightly more pronounced surface degradation in the NdFeB-400-PPS magnet.

**Figure 6:**
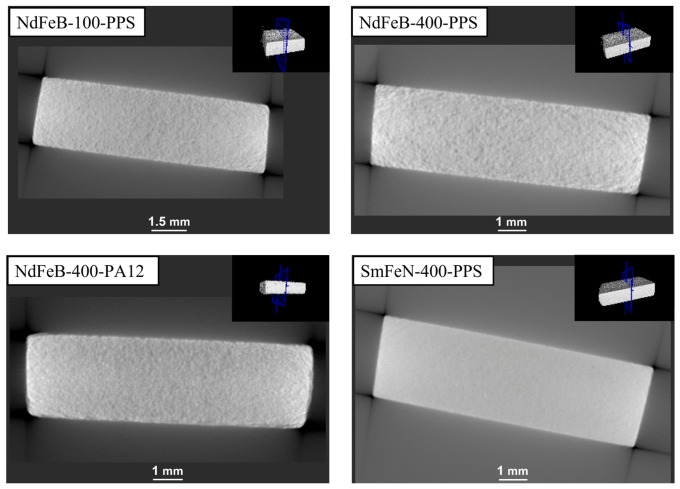
HRXCT images of Nd–Fe–B and Sm–Fe–N polymer-bonded magnets after the bulk corrosion test. The BCT test conditions: 100% RH, 120 ± 2 °C, 2 bar, 96 h. The insets show the positions of the cross-sectional planes of the magnets (indicated in blue).

HRXCT scans were performed to assess whether the corrosion extended into the interior of the magnets or remained confined to the surface (Figure 6). The HRXCT images revealed no internal damage, indicating that the corrosion was mostly superficial. Additionally, the HRXCT analysis showed that the Sm–Fe–N magnet had the most homogeneous internal structure, whereas the Nd–Fe–B magnet exhibited higher porosity. This structural difference may explain the improved corrosion resistance of the Sm–Fe–N magnets under specific conditions.

**Figure 7:**
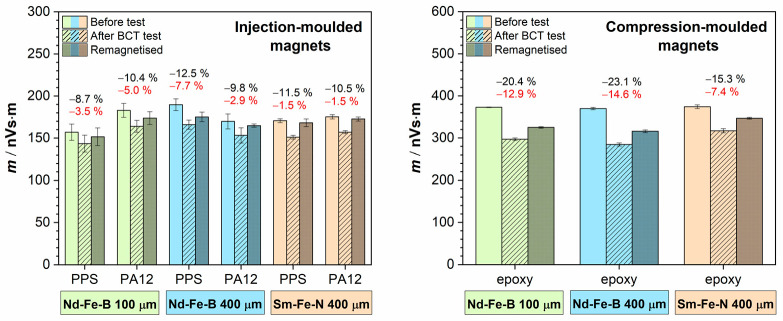
Magnetic dipole moment measurements of Nd–Fe–B and Sm–Fe–N polymer-bonded magnets before and after the bulk corrosion test and after remagnetisation of the samples. The BCT test conditions: 100% RH, 120 ± 2 °C, 2 bar, 96 h. Percentages written in black denote values of magnetic dipole moment after the bulk corrosion test, and those written in red denote values of magnetic dipole moment after the subsequent remagnetisation.

Magnetic dipole moment measurements after the corrosion test (shown as black percentages in Figure 7) revealed a decrease in the magnetic moment for all samples, including both injection-moulded and compression-moulded magnets. The reduction was lower for the injection-moulded magnets and did not exceed 13%, whereas for the compression-moulded magnets, it exceeded 15% and reached up to 23%. To evaluate the extent of damage, the samples were remagnetised after the test, and the magnetic dipole moment was measured again (shown as red percentages in Figure 7).

Although an increase in the magnetic moment was observed in all samples after remagnetisation, the values did not return to those measured before the corrosion test. This indicates irreversible corrosion-induced damage, resulting in a permanent reduction in magnetic properties. Furthermore, the injection-moulded Sm–Fe–N magnets exhibited lower losses in magnetic properties, of only 1.5%, compared to the injection-moulded Nd–Fe–B magnets, which showed losses of 3–5% for the samples with 100 µm magnetic powder and 3–8% for those with the 400 µm magnetic powder. These results confirm the superior corrosion resistance of the Sm–Fe–N magnets and support the trends observed in the electrochemical measurements. The compression-moulded magnets exhibited substantially higher permanent losses in magnetic properties. However, the Sm–Fe–N compression-moulded magnet still performed better than its Nd–Fe–B counterparts, showing a loss of up to approximately 7%. For the Nd–Fe–B compression-moulded magnets, the magnet containing 400 µm magnetic powder experienced greater corrosion than that with the 100 µm magnetic powder, exhibiting a loss of up to almost 15%, whereas the latter showed a loss of up to 13%. Although this difference is minor, it is opposite to the trend observed in the electrochemical measurements. This discrepancy may be attributed to the differences in testing conditions, as the BCT test, in addition to moisture exposure, involves elevated temperatures that promote magnet degradation through thermal oxidation.

The observed difference in the loss of magnetic properties between the injection- and compression-moulded magnets is attributed to the higher magnetic powder content in the latter, which renders them more susceptible to corrosion.

#### 3.3.2. Thermal Shock Test

**Figure 8:**
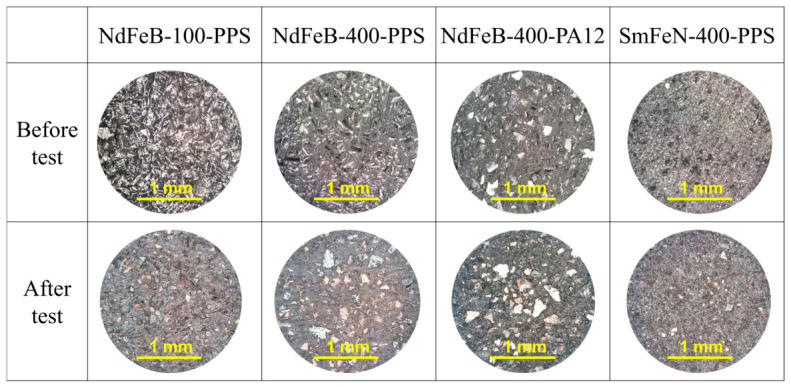
Optical microscopy images of Nd–Fe–B and Sm–Fe–N polymer-bonded magnets before and after the thermal shock test, taken in the bright-field mode at a total magnification of 100×. The thermal shock test conditions: −40/+140 °C, 30 min, 600 cycles.

**Figure 9:**
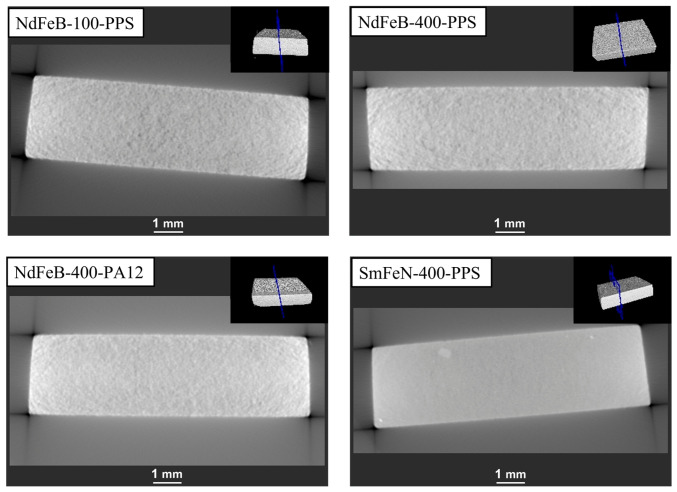
HRXCT images of Nd–Fe–B and Sm–Fe–N polymer-bonded magnets after the thermal shock test. The thermal shock test conditions: −40/+140 °C, 30 min, 600 cycles. The insets show the positions of the cross-sectional planes of the magnets (indicated in blue).

The thermal shock test was performed to evaluate the ability of the Nd–Fe–B and Sm–Fe–N polymer-bonded magnets to withstand rapid and extreme temperature changes. The optical microscopy images of the NdFeB-100-PPS, NdFeB-400-PPS, NdFeB-400-PA12, and SmFeN-400-PPS magnets (Figure 8) revealed no visible surface changes after the test. Similarly, the HRXCT images showed no internal damage, such as cracking, within the magnet structures (Figure 9).

**Figure 10:**
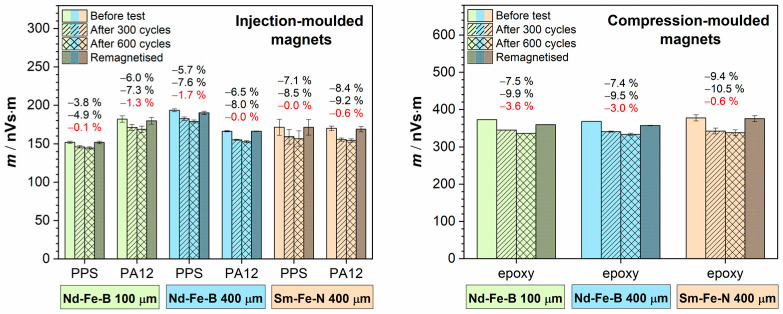
Magnetic dipole moment measurements of Nd–Fe–B and Sm–Fe–N polymer-bonded magnets before the thermal shock test, after 300 and 600 cycles, and after remagnetisation of the samples. The thermal shock test conditions: −40/+140 °C, 30 min, 600 cycles. Percentages written in black denote values of magnetic dipole moment after the thermal shock test, after 300 (first row) and 600 (second row) cycles, respectively, and those written in red after the subsequent remagnetisation.

Measurements of the magnetic dipole moment after 300 cycles (the first set of black percentage values in Figure 10) indicated a decrease in the magnetic moment of approximately 4–8% for the injection-moulded magnets, depending on the sample, whereas the compression-moulded magnets exhibited a 7–9% reduction. After 600 cycles (the second set of black percentage values in Figure 10), the injection-moulded magnets showed an additional decrease in the magnetic moment of up to approximately 9%, while the compression-moulded magnets exhibited a reduction of up to nearly 11%. However, after remagnetisation of the samples and subsequent remeasurement of the magnetic dipole moment (red percentage values in Figure 10), the Sm–Fe–N injection-moulded magnets fully recovered their original magnetic performance, while the Nd–Fe–B injection-moulded magnets nearly recovered, showing final losses of 0–2%.

The compression-moulded Sm–Fe–N magnet also regained its initial value, whereas the compression-moulded Nd–Fe–B magnets showed partial recovery, with residual losses of less than 4%, but with no visible signs of damage (Figure 8 and Figure 9). These results suggest that the loss of magnetic properties following thermal shock, in which the samples were subjected to rapid cycling between high and low temperatures, is reversible and that the magnets are thermally stable, do not crack under sudden temperature changes, and do not suffer permanent degradation of magnetic properties, unlike in the BCT, where corrosion causes irreversible losses.

## 4. Conclusions

The electrochemical behaviour of the injection- and compression-moulded Nd–Fe–B and Sm–Fe–N magnets was investigated using potentiodynamic polarisation measurements in various electrolytes with pH values from 1.8 to 12.8. The influence of magnetic powder particle size (100 µm and 400 µm), magnetic powder type (Nd–Fe–B and Sm–Fe–N), and polymer binder (PPS and PA12) on the corrosion behaviour of the magnets was examined. Electrochemical measurements indicated that the Sm–Fe–N magnets exhibited slightly better corrosion resistance than the Nd–Fe–B magnets, irrespective of the polymer binder used. The influence of magnetic powder particle size was more pronounced in the compression-moulded magnets, with the 100 µm powders showing lower corrosion resistance, likely due to their larger specific surface area relative to the 400 µm powders. The influence of the polymer binder (PPS or PA12) on the corrosion behaviour was shown to be insignificant under the tested conditions.

The bulk corrosion test and the thermal shock test were performed on the magnets to evaluate their corrosion resistance under elevated temperature and humidity conditions and their ability to withstand rapid temperature changes, respectively. In the BCT, all magnets exhibited visible surface corrosion, but HRXCT imaging confirmed that the damage did not extend into the magnets’ interiors. The compression-moulded magnets, particularly the Nd–Fe–B magnets, showed greater permanent reductions in magnetic dipole moment (up to 15%). In contrast, the thermal shock test caused temporary reductions in magnetic performance, with no visible surface or internal damage observed by optical microscopy and HRXCT. After remagnetisation, the injection-moulded magnets recovered their initial magnetic properties. The compression-moulded magnets also showed substantial recovery, although some Nd–Fe–B samples retained minor residual losses. These results indicate the thermal stability of the injection- and compression-moulded Nd–Fe–B and Sm–Fe–N magnets under rapid temperature changes.

Overall, the findings emphasise the importance of magnetic powder type and size, polymer fraction, and manufacturing method in shaping the corrosion resistance and environmental reliability of polymer-bonded magnets, with Sm–Fe–N magnets and injection-moulded composites showing the most robust performance. The combination of corrosion resistance and environmental stability makes these magnets well-suited for applications in automotive components, sensors, and electric motors exposed to demanding environmental conditions.

## Figures and Tables

**Table 1:** List of the Nd–Fe–B and Sm–Fe–N polymer-bonded magnets used in this study.

Magnetic Powder Type	Magnetic Powder Particle Size	Polymer Binder	Production Process	Abbreviation
Nd–Fe–B	100 μm	PPS	injection moulding	NdFeB-100-PPS
		PA12	injection moulding	NdFeB-100-PA12
		epoxy	compression moulding	NdFeB-100-epoxy
Nd–Fe–B	400 μm	PPS	injection moulding	NdFeB-400-PPS
		PA12	injection moulding	NdFeB-400-PA12
		epoxy	compression moulding	NdFeB-400-epoxy
Sm–Fe–N	400 μm	PPS	injection moulding	SmFeN-400-PPS
		PA12	injection moulding	SmFeN-400-PA12
		epoxy	compression moulding	SmFeN-400-epoxy

**Table 2:** EDS analysis of the mean composition of Nd–Fe–B MQP-B+ (100 μm), Nd–Fe–B MQP-B+ (400 μm), and Sm–Fe–N SFN3H (400 μm) magnetic powders corresponding to Figure 1a–c. An extended EDS analysis of spectra 1–12 is given in Table S1.

Element	Composition (at. %)		
	Nd–Fe–B MQP-B+ (100 μm) Magnetic Powder	Nd–Fe–B MQP-B+ (400 μm) Magnetic Powder	Sm–Fe–N SFN3H (400 μm) Magnetic Powder
C	43.8	55.7	26.0
O	4.6	5.1	6.4
Fe	24.9	20.4	52.4
Nd	4.3	3.5	-
B	12.0	12.2	-
Sm	-	-	5.6
N	-	-	6.3
Co	1.9	1.6	1.5
Al	8.5	1.4	<0.5
Ga	-	-	<1
Zr	-	-	<1
Zn	-	<0.5	-

## Data Availability

All data and materials are available on request from the corresponding author. The data are not publicly available due to ongoing research using part of the data.

## Supplementary Materials

The following supporting information can be downloaded at https://www.mdpi.com/article/10.3390/polym18091123/s1, Figure S1: Graphs illustrating the influence of (a–c) magnetic powder type, (d–f) magnetic powder size, and (g–i) polymer binder on the corrosion behaviour of Nd–Fe–B and Sm–Fe–N polymer-bonded magnets, showing the dependence of *E*_corr_ on pH. The data were obtained from the PDP measurements and correspond to the mean values of several repeated measurements, with error bars representing the standard deviations; Figure S2: Open-circuit potential of the NdFeB-400-PPS and SmFeN-400-PPS magnets in different electrolytes, monitored during 1 h at room temperature; Table S1: EDS analysis of Nd–Fe–B MQP-B+ (100 μm), Nd–Fe–B MQP-B+ (400 μm), and Sm–Fe–N SFN3H (400 μm) magnetic powders corresponding to Figure 1a–c analysed at different sites.
