# Supplementary material for: Corrosion Behaviour of Injection- and Compression-Moulded Nd–Fe–B and Sm–Fe–N Magnets with Different Polymer Binders
Source: Polymers (Basel). 2026 May 2;18(9):1123. doi: 10.3390/polym18091123 (PMC13165790; doi:10.3390/polym18091123)
Supplement: Supplementary file 1 [file polymers-18-01123-s001.zip › polymers-4259487-supplementary.pdf]

Corrosion behaviour of injection- and compression-moulded Nd–Fe–B and Sm–Fe–N magnets with different polymer binders

Nikolina Lešić [1,2], Nataša Kovačević [3] and Ingrid Milošev [1,2,*]

Supplementary material

Table S1. EDS analysis of Nd–Fe–B MQP-B+ (100 μm), Nd–Fe–B MQP-B+ (400 μm), and Sm–Fe–N SFN3H (400 μm) magnetic powders corresponding to Figures 1a–c analysed at different sites.

**Element**	**Composition (at. %)**			
	**Nd–Fe–B MQP-B+ (100 μm) magnetic powder**			
	Spectrum 1	Spectrum 2	Spectrum 3	Spectrum 4
B	11.1	13.5	12.9	10.5
C	42.0	41.8	49.7	41.9
O	4.6	4.5	4.6	4.6
Al	6.9	8.2	10.5	8.5
Fe	28.5	25.8	17.7	27.6
Co	2.1	1.8	1.3	2.3
Nd	4.8	4.3	3.2	4.7
	**Nd–Fe–B MQP-B+ (400 μm) magnetic powder**			
	Spectrum 5	Spectrum 6	Spectrum 7	Spectrum 8
B	13.4	11.8	12.8	10.8
C	57.9	54.2	54.3	56.5
O	4.5	5.5	4.8	5.8
Al	1.4	1.5	1.4	1.4
Si	–	0.1	–	–
Fe	18.3	21.7	21.3	20.5
Co	1.2	1.7	1.7	1.5
Zn	0.2	–	–	–
Nd	3.0	3.6	3.7	3.5
	**Sm–Fe–N SFN3H (400 μm) magnetic powder**			
	Spectrum 9	Spectrum 10	Spectrum 11	Spectrum 12
C	25.1	20.3	25.3	33.2
N	9.2	4.1	4.9	6.8
O	6.2	5.6	6.0	7.8
Al	–	1.1	–	0.8
Fe	51.6	57.9	54.7	45.2
Co	–	3.2	2.6	–
Ga	1.2	0.5	0.5	0.9
Zr	0.7	0.6	0.6	0.8
Sm	6.0	6.8	5.2	4.5

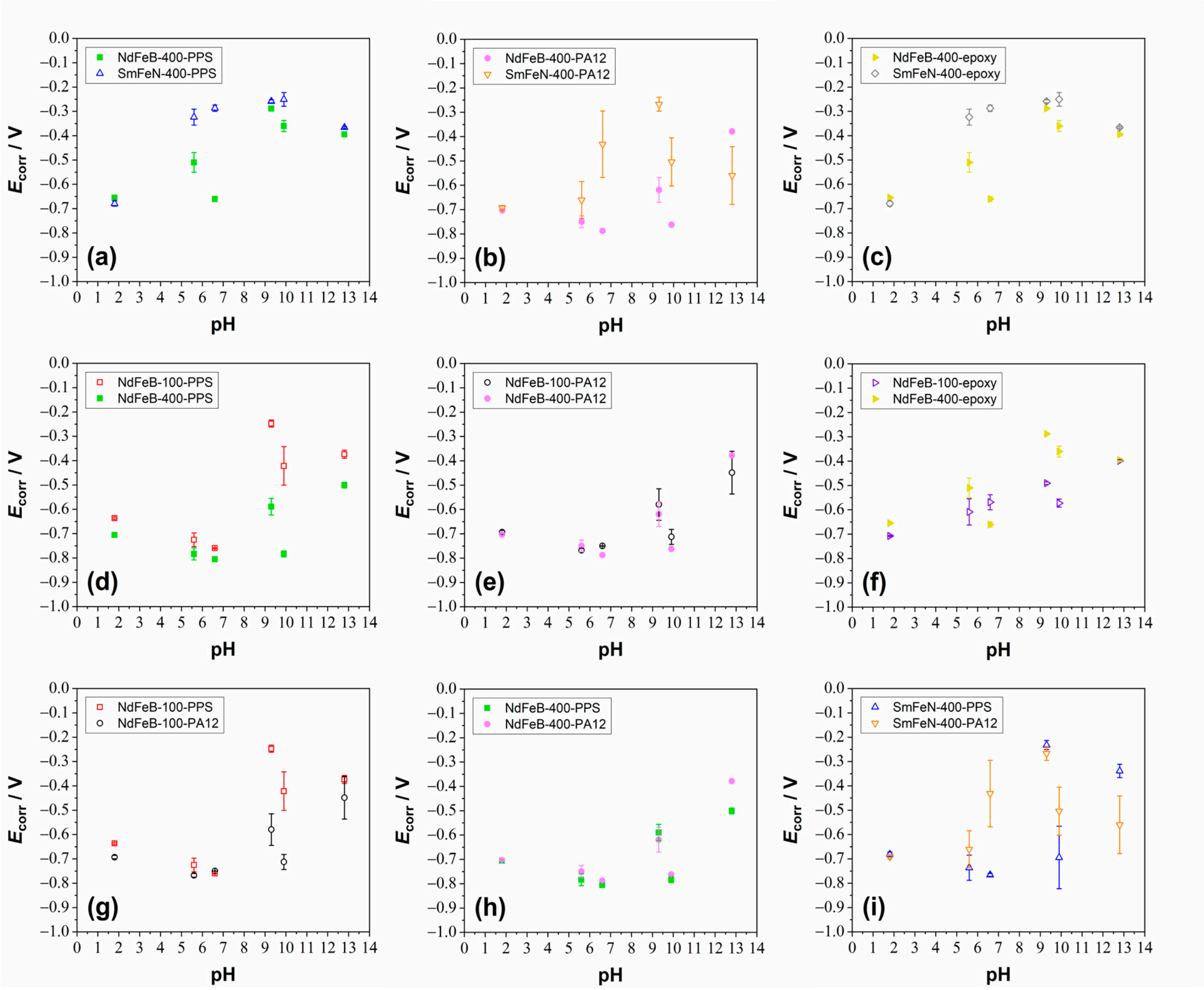

Figure S1. Graphs illustrating the influence of (**a–c**) magnetic powder type, (**d–f**) magnetic powder size, and (**g–i**) polymer binder on the corrosion behaviour of Nd–Fe–B and Sm–Fe–N polymer-bonded magnets, showing the dependence of E_{corr} on pH. The data were obtained from the PDP measurements and correspond to the mean values of several repeated measurements, with error bars representing the standard deviations.

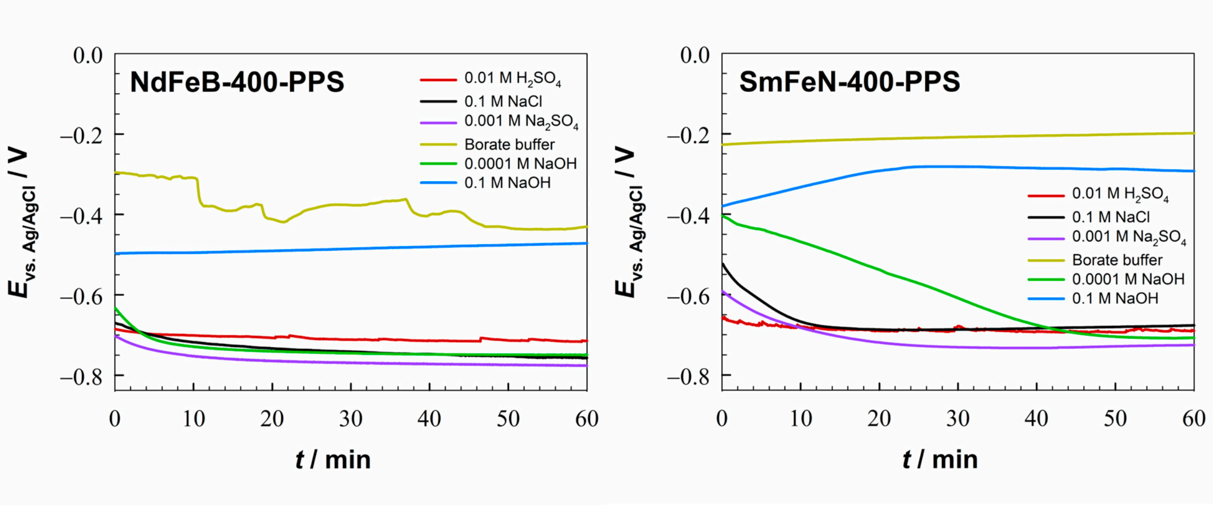

Figure S2. Open circuit potential of the NdFeB-400-PPS and SmFeN-400-PPS magnets in different electrolytes, monitored during 1 h at room temperature.
